# Supplementary material for: Systematic Cardiovascular Screening in Olympic Athletes before and after SARS-CoV-2 Infection
Source: J Clin Med. 2022 Jun 17;11(12):3499. doi: 10.3390/jcm11123499 (PMC9224878; doi:10.3390/jcm11123499)
Supplement: Supplementary file 1 [file jcm-11-03499-s001.zip › jcm-1751585-supplementary.pdf]

Supplementary matherial

Title. Systematic cardiovascular screening in Olympic athletes before and after SARS-CoV-2 infection.

The following data have been provided as supporting material due to limited space in the main manuscript. Although not essential for the understanding of the paper, the authors felt that these will complement the main manuscript.

Details on the cardiovascular screening protocol

Blood tests included: full blood count, alanine (ALT) and aspartate (AST) aminotransferase, gamma-glutamyl transferase (GGT), creatinine, creatinine phosphokinase (CPK), high-sensitivity Troponin (hs-Trop), lactic acid dehydrogenase (LDH), prothrombin time (PT), partial thromboplastin time (PTT), international normalized ratio (INR), protein electrophoresis, D-dimer, C reactive protein (CRP), ferritin, interleuchin-6 (IL-6), urine examination.

Pulmonary function tests (PFTs): Forced Vital Capacity (FVC), Forced Expiratory Volume in 1 second (FEV1) and the FEV1/FVC ratio were measured and expressed as percent of the predicted, and compared with values derived from normal subjects of the same age, gender, height, weight and ethnicity.

12-lead resting electrocardiogram (ECG) was recorded with the subject in supine position during quiet respiration, at 25 mm/s, using a Cardioline ClickECG (Cardioline, Italy).

Transthoracic Echocardiogram (TTE) was performed using Philips Epiq7 (Philips Medical Systems, Andover, Massachusetts) equipped with an S3 probe (2 to 4 MHz).

Cardiopulmonary exercise test (CPET) was a maximal, symptom-limited continuous ramp CPET, using a cycle ergometer (MORTARA) connected with Quark CPET (COSMED). A COSMED V2 silicon Oro-Nasal Mask was worn by each athlete during testing. Each test included the recording

of cardiac and ventilator parameters for about 1 minute at rest; subsequently the ramp protocol was started until the exhaustion and followed by 4 minutes of recovery. The following parameters were collected: Maximal Heart Rate (HR max), Maximal Systolic and Diastolic Blood Pressure (SBP and DBP max), Maximal workload (Watt max), Maximal Ventilation (VE max), Maximal Oxygen Uptake (VO2 max), Ventilator Efficiency Slope (VE/VCO2 slope), Oxygen Pulse (VO2/HR) and Peak Respiratory Exchange Ratio (RER max). The peak VO2 was the highest VO2 during a 10-s interval obtained at the end of exercise. The Lactate Threshold (LT) was determined using both the V-slope and the ventilatory equivalent for O2 graphs. Oxygen saturation measures were collected with a digital pulse oximeter (KAARSEN) at rest, at maximum physical effort and at the end of the recovery phase. Resting and peak blood pressure, any supraventricular and/or ventricular arrhythmias, ST/T changes and symptoms during the exercise were recorded.

CMR was performed by a 1.5 T scanner (GE Signa) and comprised long and short-axis cine, T2-weighted images in short axis for edema, native T1 Mapping by MOLLI (with 5(3)3 sampling) sequences and T2 Mapping (based on multi-echo fast-spin-echo - MEFSE - sequence with 4 echo times-TEs) in three short axis (base-mid- apex) and three long-axes. After an intravenous contrast agent dose of 0.1 mmol/kg Gadolinium-DOTA (Dotarem, Guerbet-France) post-contrast breath-hold inversion recovery prepared T1-weighted gradient echo sequence was acquired in long and short axis for late gadolinium enhancement (LGE) evaluation. Post-contrast T1 mapping (with 4(1)3(1)2 sampling) was acquired for ECV quantification.
